# Opioid Misuse: A Review of the Main Issues, Challenges, and Strategies

**DOI:** 10.3390/ijerph191811754

**Published:** 2022-09-17

**Authors:** Helena Biancuzzi, Francesca Dal Mas, Valerio Brescia, Stefano Campostrini, Marco Cascella, Arturo Cuomo, Lorenzo Cobianchi, Ander Dorken-Gallastegi, Anthony Gebran, Haytham M. Kaafarani, Franco Marinangeli, Maurizio Massaro, Angela Renne, Giacomo Scaioli, Rym Bednarova, Alessandro Vittori, Luca Miceli

**Affiliations:** 1 Department of Clinical and Experimental Pain Medicine, Istituto di Ricovero e Cura a Carattere Scientifico—IRCCS Centro di Riferimento Oncologico—CRO of Aviano, 33081 Aviano, Italy; 2 Department of Management, Ca’ Foscari University of Venice, 30100 Venice, Italy; 3 Department of Management, University of Turin, 10134 Turin, Italy; 4 Department of Finance, Wrocław University of Economics and Business, 53-345 Wrocław, Poland; 5 Department of Economics, Ca’ Foscari University of Venice, 30100 Venice, Italy; 6 Division of Anesthesia and Pain Medicine, Istituto Nazionale Tumori, Istituto di Ricovero e Cura a Carattere Scientifico—IRCCS, Fondazione Pascale, 80131 Naples, Italy; 7 Department of Clinical, Diagnostic and Pediatric Sciences, University of Pavia, 27100 Pavia, Italy; 8 General Surgery Department, Istituto di Ricovero e Cura a Carattere Scientifico—IRCCS Policlinico San Matteo Foundation, 27100 Pavia, Italy; 9 Division of Trauma, Emergency Surgery and Surgical Critical Care, Massachusetts General Hospital, Boston, MA 02114, USA; 10 Harvard Medical School, Boston, MA 02115, USA; 11 Department of Life, Health and Environmental Sciences, University of L’Aquila, 67100 L’Aquila, Italy; 12 Department of Public Health Sciences and Pediatrics, University of Turin, 10126 Turin, Italy; 13 Department of Pain Medicine, Hospital of Latisana, 33053 Latisana, Italy; 14 Department of Anesthesia and Critical Care, ARCO, Ospedale Pediatrico Bambino Gesù, Istituto di Ricovero e Cura a Carattere Scientifico—IRCCS, 00165 Rome, Italy

**Keywords:** opioid misuse, opioid epidemic, opioid prescription, public health, literature review, social impacts

## Abstract

In the United States, from 1999 to 2019, opioid overdose, either regularly prescribed or illegally acquired, was the cause of death for nearly 500,000 people. In addition to this pronounced mortality burden that has increased gradually over time, opioid overdose has significant morbidity with severe risks and side effects. As a result, opioid misuse is a cause for concern and is considered an epidemic. This article examines the trends and consequences of the opioid epidemic presented in recent international literature, reflecting on the causes of this phenomenon and the possible strategies to address it. The detailed analysis of 33 international articles highlights numerous impacts in the social, public health, economic, and political spheres. The prescription opioid epidemic is an almost exclusively North American problem. This phenomenon should be carefully evaluated from a healthcare systems perspective, for consequential risks and harms of aggressive opioid prescription practices for pain management. Appropriate policies are required to manage opioid use and prevent abuse efficiently. Examples of proper policies vary, such as the use of validated questionnaires for the early identification of patients at risk of addiction, the effective use of regional and national prescription monitoring programs, and the proper dissemination and translation of knowledge to highlight the risks of prescription opioid abuse.

## 1. Introduction and Background Literature

Opioids are a class of substances that inhibit the transmission of painful stimuli [1]. They are characterized by a strong analgesic effect. Opioids may be legally prescribed in the form of opiates such as codeine, morphine, semisynthetic opioids such as oxycodone, synthetic opioids such as fentanyl [2], and there are also illegal forms such as heroin [1].

From 1999 to 2019 in the United States, opioid overdose from both regularly prescribed and illegally acquired drugs was the cause of death for nearly 500,000 people [3]. These deaths did not occur at a consistent rate over time, but gradually increased during the period. From 1999 to 2018, opioid overdoses quadrupled [4]. This increase in opioid overdose deaths is delineated in three distinct waves [5,6]: the first from legally prescribed and manufactured opioid drugs; the second from heroin; and the third by illicitly manufactured synthetic opioids.

The first wave occurred in the 1990s with the increase in opioid prescriptions and prescription-related deaths from overdoses of natural and semi-synthetic opioids [7]. The second wave conventionally started in 2010 and is characterized by a rapid rise in deaths from heroin overdoses [8]. The latest wave occurred in 2013 with a significant increase in overdose deaths involving synthetic opioids, mainly illicitly produced fentanyl [9,10,11].

In patients undergoing treatment with opioid drugs, excluding cases of maladministration or self-administration, we must distinguish two conditions of misuse or abuse for two classes of substances. The two forms of abuse are: (1) abuse by a patient newly addicted, following prescription with continuous use of opioids at non-prescription dosages; and (2) abuse by a patient previously addicted to the use of opioids.

For the classes of substances, the following are recognized: (1) prescribed opioid drugs; and (2) illegally acquired opioid drugs.

There are various possible reasons underlying the opioid epidemic. As evidenced in recent literature, opioid prescriptions for pain relief have increased [5,6,12,13,14,15]. This correlates to the increasing focus in the medical field on pain management [16,17]. However, the deaths in the latest wave of the opioid epidemic are attributed to a population between 25 and 35 years of age. This is a markedly different population than typical pain medication users, who are usually older. Furthermore, the greater accessibility to opioid substances has led to potentially high-risk contextual conditions [3]. Patients may take more doses or for a greater duration than prescribed, use drugs prescribed to others, or obtain substances illegally without a legitimate prescription [3,18]. This scenario makes it clear that opioid misuse is now considered an epidemic in the United States [19]. According to the literature, the phenomenon has various impacts, which affect the medical sphere (in terms of prescription, clinical consequences, and comorbidities) but also the social and financial ones, involving the patients, the population, and the healthcare system [20,21,22].

The opioid epidemic is a theme that has recorded over 4350 contributions on the PubMed scientific dataset in the last ten years, with a growing trend that has seen 1117 articles in the year 2020 alone.

In countries other than the United States, opioids are underprescribed [23,24]. Although the opioid status in these countries differs from that of North America, there remains a need to assess prevention strategies for the opioid epidemic. For patients at a high risk of addiction, it is necessary to be able to assess the exposure factors prior to prescribing opioids [23,24,25]. The potential increase in the illicit use of opioids must also be monitored in areas other than pain management. Additionally, specific attention should be paid to the growth in the use of other illicit substances, which are often consumed along with opioids [26].

Starting from the premises above, this literature review aims to seek international evidence on opioid abuse to prevent and combat opioid addiction, especially as a monitor to those countries that find themselves in an underprescription stage.

The article offers a critical analysis of the literature in the context of the opioid epidemic, suggesting practice and research avenues for health professionals, decision makers, and scholars.

## 2. Methodology

A structured literature review [27] was conducted using the Scopus and Web of Science datasets [28]. A first preliminary research protocol was defined to document the procedures for developing the literature review. The structured reviews aim to critically investigate (critique) an existing field of knowledge to provide an overview of developments and offer new in-depth research paths and operational guidelines. The formalization of the research protocol helped to identify the central question to be investigated, which was defined as follows:

*Research question (RQ): What are the trends and consequences of opioid misuse analyzed in the recent literature?*

**Figure 1:**
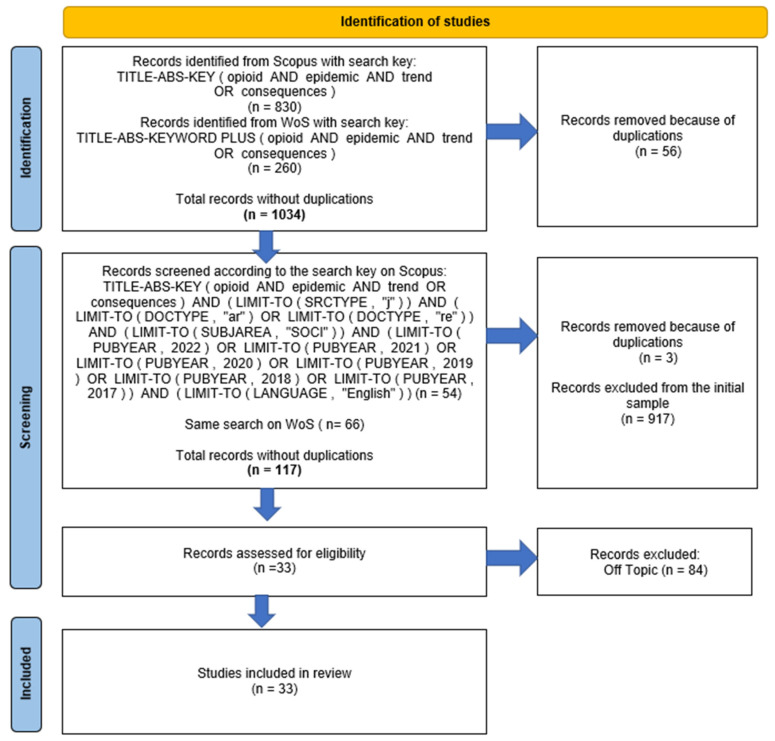
Process of article selection using the PRISMA methodology. Source: Authors’ elaboration following Page et al. [30].

The first search string used concerned the words “Opioid” and “Epidemic” in addition to “Trend” and “Consequences”, searched in the Abstract, Title, and Keywords (Keywords Plus on Web of Science); this led to 830 documents results on Scopus and 260 on Web of Science. Fifty-six duplicates were found and removed, leading to 1034 total results. The research was then refined to include only articles, journals, and reviews in English, published between 2017 and 2022 in both datasets. The new analysis yielded 54 results on Scopus and 66 on Web of Science, leading to 117 total documents excluding duplicates. All the contributions were found, and all the abstracts were read and analyzed by two authors (HB and FD) to ensure eligibility. Thirty-three papers were eligible after analysis and focused on the topic under investigation. Figure 1 below summarizes the process of selection of the contributions to be included in the sample according to the PRISMA methodology [29,30]. The final sample concerned the articles listed in Table 1. The selected papers were coded and analyzed using the Nvivo software (version 12, QSR International, Doncaster, Australia).

## 3. Results

The selected articles were analyzed using a coding framework with nodes derived from previous studies in knowledge management in the public sector [59] and healthcare management [60,61,62].

Specifically, some of the nodes borrowed from previous studies concern the authors’ classification, division of academic and non-academic/practitioners, geographical areas of the research, research methodology, level of impact, and stakeholders involved. Other nodes related to the study objective were added to the framework with an open-coding approach [27]. In particular, after assessing and reading the papers belonging to the sample, the authors decided to map the type of epidemic and the eventually recommended strategies. Table 2 reports the framework used for coding the selected articles and the final results.

Considering the 33 articles analyzed, just over half were published by academic authors, namely those physicians or researchers holding (also) an academic position. All the authors’ affiliations were carefully checked to understand the eventual academic engagement and position. When the job role was not clear (e.g., in a university hospital), the names were searched online to understand the eventual academic engagement. About a third of the outputs were generated in a collaboration between academic authors and professionals, namely physicians, without any academic roles. Five contributions were generated only by the work of practitioners without involving people from the academia.

Three-quarters of the contributions analyzed are quantitative studies (27 contributions), of which five are survey-based. The remaining quarter (6 references) is qualitative, including two literature reviews. The reference area is practically mono-geographic: 28 of the 33 contributions refer to studies conducted in the United States, with one in Canada. The remaining four papers are not linked to a specific location.

Concerning the topics, the twin epidemic is the most discussed, with 8 references. The twin epidemic refers to the phenomenon when a different addiction is accompanied by that of opioids. Another topic focuses on the effects on children of addicted mothers (5 references). These children face the consequences such as post-birth care due to neonatal abstinence syndrome. Three contributions deal with the effects when it comes to First Aid/Emergency Rooms (ER) access. The papers underline how ER accesses are higher in the places analyzed where the use of opiates is widespread. Nineteen of the contributions, on the other hand, are placed in a more generic area, not going into the specifics of a particular sector.

Concerning the most relevant impacts, the analysis allowed to group such outcomes into three distinct categories: the social, health, and public health sphere; the medical sphere; and the economic, justice and political sphere. Influences from the social and public health sphere include the illegal use of methamphetamine and fentanyl [6,49], and the rise in the number of deaths [14,39] and homicides [50,51]. Effects from a medical perspective involve infectious complications [7] and neonatal abstinence syndrome [36]. Outcomes relating to the economy, justice, and politics lead to an increase in healthcare costs [5,58] and a rise in violence [36,48,51]. Table 3 below shows some of the most pertinent subgroups, the bibliographic references taken from the sample, and some contents extracted from the analyzed articles for each type of impact.

As for the stakeholders, Table 4 reports the main stakeholders identified in the analyzed articles. Prevalent stakeholders include patients, the justice system, the health system, pharmacies, and minors, defined as the children of addicted patients. For each of such stakeholders, the literature underlines some relevant issues. Such issues include the dangers associated with addiction, the effects on social life, the need to acquire financial resources even illegally, the complex professional relationship between physicians and pharmacists concerning the required prescriptions, and the complicated family situations that may also impact the children of addicted patients, among others.

Regarding the numerous strategies recommended to deal with the phenomenon, among the most cited, it emerges the clear urge to enhance training and information for both healthcare providers and patients [5,12,14,31,41,45,49,52,53,54,57]. This is essentially related to the recommendation to prevent rather than implement subsequent containment and corrective action [12,31,36,40,42,46,54,58].

In the articles, testimonies also stress the worrying extent of the phenomenon, as in the case of Scorsone and colleagues [54], who cites regarding a patient addicted to opioids:

*“I always said I would never do that [share needles]. (…) and then that’s when it happens. (…) This one time, I was in jail–I didn’t have anything, and I shot up in jail like an idiot. I didn’t know that everybody had hep C.”*

[54]

Other strategies suggested by multiple contributions are to implement programs to treat opioid addiction [4,5,34,40,46] and increase research, grants, and funding in the sector [36,46,48,50,56]. In addition, many contributions support the need to implement medical care [31,36,42,43,46] and increase the prescription and use of naloxone [12,31,33,42,43]. Cordes [12] reports the results of his study as follows:

*“(…) the results of this study can be used to inform counties considering implementing naloxone programs, an important medication used to reverse opiate overdose.”*

[12]

Five contributions argue that it is desirable to proceed with multidisciplinary approaches to address the problem, starting from a complete overview and joining the forces and potential of several institutions and professions [5,38,47,51,55]. The following reflections by a pharmacist and a medical manager emerge in the articles analyzed:

*“I know that I personally do my best to monitor for signs of misuse or abuse and to counsel as necessary, but I don’t have access to patient charts, histories or diagnosis or to the attention of the patient as physicians do.”*

[53]

*“(…) traditional utilization management approaches appeared to be ineffective in slowing the spread of the epidemic. We believed more could be done. We also recognized that no single entity could do this alone. We began working with many partners (…) coordinating teams internally, including our medical policy, pharmacy, and fraud teams, (…)”*

[5]

## 4. Discussion

The literature on opioid use and its consequences is mainly concentrated in the United States. This may lead to the conclusion that the opioid epidemic is primarily confined to this geographical area [63]. The reasons may lie in the characteristics of the health system, with predominantly private health care providers, and the legislation of the system. Opioid therapies are among the cheapest and most immediate for pain therapy compared to other treatments such as physiotherapy, as can be seen in other countries—e.g., Italy—by crossing the rates for specialist outpatient services and the costs of opioid drugs. Still, abuse and addiction can lead to numerous social, health, and legal implications.

Understanding the developments, consequences, and containment strategies of this phenomenon is relevant to all countries in a phase of underprescription. The North American epidemic and its impacts serve as a warning for implementing policies to avoid the consequences of inappropriate opioid use.

Concerning the analysis of the results acquired in the selected literature and future lines of study, the articles in the sample show that the number of collaborations between clinicians and scholars is significant. This brings out an essential collaborative effort between academia and professionals in the field. Furthermore, the research methods used vary, and this denotes the possibility of interpreting the phenomenon in a multifaced way.

Moreover, it is necessary to underline the data relating to the twin epidemic phenomenon and the numerous contributions that report adverse effects in the pediatric field.

The international literature reveals various intervention opportunities ranging from training to research, from economic support to a variation in the pharmacological approach (like in the use of naloxone). The in-depth study conducted with this review underlines the need to work further on real-world data and scientific contributions to increase the knowledge of the phenomenon. Real-world data will allow for monitoring opioid prescriptions to relate to, and compare, what has been observed in the literature. A knowledge translation approach [62] should be employed to ensure that clinicians of various specialities (including pain medicine physicians, general practitioners, oncologists, surgeons, …) can communicate with patients, also “speaking less and listening more,” [64] to detect sensitive situations in which opioids should not be prescribed or should be more strictly monitored. Moreover, patients and citizens should be aware of the consequences that may arise from addiction. Other strategies may include state-level prescription drug monitoring programs and mandatory enrollment of prescribers in these programs [65], legislative action to control first-time prescriptions of opioids to opioid-naive patients, systematic guidelines to standardize the prescription of opioids for acute pain (e.g., post-surgical pain) [66,67], and education and provision of resources for the correct disposal of unused prescription opioids to prevent diversion. Safe storage and live inventory strategies of opioid-based drugs should also be ensured in hospitals and pharmacies.

It should be noted that all these initiatives must consider the rights of patients to receive adequate analgesia and avoid chronic pain, for which opioids are particularly effective. Physicians and other healthcare professionals should balance the patients’ right to analgesia and the need to prevent the overuse and abuse of opioids [68]. As mentioned in some of the studies included in the review, it is mandatory to act on several fronts: on the one hand, to increase the knowledge, skills, and competencies of healthcare providers on the topic of “pain therapy” [69], often placed in the background of the study programs of medical and healthcare professions degrees [70]. On the other hand, reaching favorable conditions for rapid and early identification of patients who may be at risk of addiction is essential. In this regard, the implementation and dissemination of (personal) electronic health records that allow effective sharing of health data could be useful to clinicians and healthcare professionals, and should be fostered by policymakers and healthcare managers [71].

Other considerations may arise, concentrating not only on what the literature reports, but what is missing, such as prevention strategies. In the 1990s, knowledge translation was employed to make young adults and kids aware of the risks and dangers of heroin. However, these practices are no longer in use. Involving adolescents and young adults in the dialogue with dedicated programs may be a strategy to educate them on the dangers of opioid misuse. Moreover, the internet now represents a source of information, but it also offers opportunities to acquire illegal drugs from online stores, or legal ones without a valid prescription [63]. Opioid-based medications could be easier to obtain. For this reason, competent authorities should monitor the web and these online pharmacies to avoid such activity.

Finally, when referring to the main stakeholders, most of the papers included in the sample do not report the general population. Indeed, the literature focuses on patients, clinicians, or health institutions trying to prevent or fight the phenomenon. As mentioned above, patients may eventually need opioids for pain relief because of a chronic disease or an acute phase. Therefore, education to understand the risk of addiction and the need to follow prescriptions may be relevant. As for children and young adults, prevention campaigns may represent an option for the general population to be fully aware of the phenomenon. According to the main theories about change in human behaviours (e.g., Rogers’ theory on the diffusion of innovation [72]), it would be necessary to act by increasing knowledge on opioid consumption, promoting the acquisition of skills and competencies, and fostering “enabling” factors (e.g., environmental determinants) that can favor a balanced consumption of opioids by the population [73,74]. This implies a multidisciplinary approach that involves clinicians, health promotion professionals, researchers, policymakers, and other meaningful stakeholders, as stated by the Centers for Disease Control and Prevention, that in 2019 developed a “vademecum” on how to prevent opioid overdoses [75]. The main goal is to avoid “blaming the victims”, by considering opioid abuse as more than individual responsibility and as the consequence of several multifaceted determinants.

### Limitations

Like all pieces of research, ours is not without limitations. Although the primary scientific datasets have been investigated according to a rigorous methodology, some relevant contributions may have been excluded. The topic is immense, and further efforts could include enlarging the number of enquired datasets and keywords. Machine Learning algorithms could also be used to select the most relevant contributions and ensure better coverage. Moreover, given the importance of the topic to the general population and healthcare system in several countries, other sources could have been added, including reports, guidelines, and recommendations from primary scientific societies, public health bodies, and other non-profit organizations devoted to the cause. Moreover, the multifaced and complex dynamics of the phenomenon require constant updating since results may not be applicable to other geographical contexts or situations. The analysis of the so-called “grey” literature and the investigation of country-specific factors may lead to new research avenues for scholars and practitioners like healthcare policymakers engaged in the topic.

## 5. Conclusions

Health decision makers from all over the globe can benefit from the studies on the US opioid epidemic to learn and be inspired and warned by the American experience, its risks, costs, and outcomes. Our literature review has allowed detection of the principal risks and dangers and identified feasible strategies to limit and prevent the phenomenon, including training, dedicated research funds, alternative drugs, and other therapies. Considering the adverse outcomes for the population and the healthcare system in cases of overdose, additional measures should be employed for the early identification of patients at risk of addiction. From such a perspective, the diffusion of personal health electronic records is crucial to ensure effective knowledge transfer among all healthcare professionals taking care of a patient who could be at higher risk of becoming addicted.

Prevention is another relevant strategy to actively involve patients and citizens through adequate dissemination and translation of knowledge to highlight the risks of overdose or abuse of opioid drugs.

## Figures and Tables

**Table 1:** Papers included in the sample.

N.	Authors	Title	Journal	Reference
1	Vivolo-Kantor A.M., Seth P., Gladden R.M., Mattson C.L., Baldwin G.T., Kite-Powell A., Coletta M.A.	Vital signs: Trends in emergency department visits for suspected opioid overdoses—United States, July 2016–September 2017	Morbidity and Mortality Weekly Report	[31]
2	Fleischauer A.T., Ruhl L., Rhea S., Barnes E.	Hospitalizations for endocarditis and associated health care costs among persons with diagnosed drug dependence-North Carolina, 2010–2015	Morbidity and Mortality Weekly Report	[32]
3	Chiu A.S., Healy J.M., DeWane M.P., Longo W.E., Yoo P.S.	Trainees as Agents of Change in the Opioid Epidemic: Optimizing the Opioid Prescription Practices of Surgical Residents	Journal of Surgical Education	[14]
4	Guy G.P., Jr., Haegerich T.M., Evans M.E., Losby J.L., Young R., Jones C.M.	Vital signs: Pharmacy-based naloxone dispensing—United States, 2012–2018	Morbidity and Mortality Weekly Report	[33]
5	Lynch S., Sherman L., Snyder S.M., Mattson M.	Trends in infants reported to child welfare with neonatal abstinence syndrome (NAS)	Children and Youth Services Review	[34]
6	Strickler G.K., Kreiner P.W., Halpin J.F., Doyle E., Paulozzi L.J.	Opioid prescribing behaviors-Prescription behavior surveillance system, 11 states, 2010–2016	MMWR Surveillance Summaries	[35]
7	Graves R.L., Tufts C., Meisel Z.F., Polsky D., Ungar L., Merchant R.M.	Opioid Discussion in the Twittersphere	Substance Use and Misuse	[13]
8	Saunders J.B., Jarlenski M.P., Levy R., Kozhimannil K.B.	Federal and State Policy Efforts to Address Maternal Opioid Misuse: Gaps and Challenges	Women’s Health Issues	[36]
9	Daniulaityte R., Silverstein S.M., Crawford T.N., Martins S.S., Zule W., Zaragoza A.J., Carlson R.G.	Methamphetamine Use and Its Correlates among Individuals with Opioid Use Disorder in a Midwestern U.S. City	Substance Use and Misuse	[37]
10	Eeckhaut M.C.W., Wagner J., Neitzke-Spruill L., Walker R., Anderson T.L.	Is the gender gap in overdose deaths (Still) decreasing? an examination of opioid deaths in Delaware, 2013–2017	Journal of Studies on Alcohol and Drugs	[6]
11	Cordes J.	Spatial trends in opioid overdose mortality in North Carolina: 1999–2015	Southeastern Geographer	[12]
12	Chiarello E.	Where Movements Matter: Examining Unintended Consequences of the Pain Management Movement in Medical, Criminal Justice, and Public Health Fields	Law and Policy	[38]
13	Seltzer N.	The economic underpinnings of the drug epidemic	SSM-Population Health	[39]
14	Bushman G., Victor B.G., Ryan J.P., Perron B.E.	In Utero Exposure to Opioids: An Observational Study of Mothers Involved in the Child Welfare System	Substance Use and Misuse	[40]
15	Sud A., Doukas K., Hodgson K., Hsu J., Miatello A., Moineddin R., Paton M.	A retrospective quantitative implementation evaluation of Safer Opioid Prescribing, a Canadian continuing education program	BMC Medical Education	[41]
16	Queeneth U., Bhimanadham N.N., Mainali P., Onyeaka H.K., Pankaj A., Patel R.S.	Heroin overdose-related child and adolescent hospitalizations: Insight on comorbid psychiatric and substance use disorders	Behavioral Sciences	[42]
17	Feinglass J., Wang J.A., Ye J., Tessier R., Kim H.	Hospital Care for Opioid use in Illinois, 2016–2019	Journal of Behavioral Health Services and Research	[43]
18	Sobotka T.C., Stewart S.A.	Stereotyping and the opioid epidemic: A conjoint analysis	Social Science and Medicine	[44]
19	Aguilar-Amaya M., Gutiérrez M., Sr.	Implementing Compassion Fatigue Prevention for Lay Employees Conducting Naloxone Training: An Example from Rural Arizona	Journal of Social Work Practice in the Addictions	[45]
20	Wagner J., Neitzke-Spruill L., Donnelly E.A., O’Connell D.J., Anderson T.L.	The Current Community Context of Overdose Deaths: Relations among Drug Types, Drug Markets, and Socioeconomic Neighborhood Characteristics	Sociological Forum	[46]
21	Wentzlof C.A., Boman IV J.H., Pryor C., Hemez P.	“Kicking the Can down the Street”: Social Policy, Intimate Partner Violence, and Homicide during the Opioid Crisis	Substance Use and Misuse	[47]
22	Gollust S.E., Haselswerdt J.	A crisis in my community? Local-level awareness of the opioid epidemic and political consequences	Social Science and Medicine	[48]
23	Odusola F., Kaufman J., Turrigiano E., Aydinoglo N., Shulman M., Kidd J., Hu M.-C., Levin F.R.	Predoctoral substance use disorders curricula: A survey analysis and experiential pedagogy	Journal of Dental Education	[49]
24	Rosenfeld R., Roth R., Wallman J.	Homicide and the Opioid Epidemic: A Longitudinal Analysis	Homicide Studies	[50]
25	Testa A., Weiss D.B., Santos M.R.	Opioid mortality, public health care expenditures, and cross-national homicide rates: findings from 25 OECD countries, 2000–2017	Social Psychiatry and Psychiatric Epidemiology	[51]
26	Sud A., Hodgson K., Bloch G., Upshur R.	A Conceptual Framework for Continuing Medical Education and Population Health	Teaching and Learning in Medicine	[52]
27	Rao D., Giannetti V., Kamal K.M., Covvey J.R., Tomko J.R.	Pharmacist Views Regarding the Prescription Opioid Epidemic	Substance Use and Misuse	[53]
28	Scorsone K.L., Haozous E.A., Hayes L., Cox K.J.	Ending the Chase: Experiences of Rural Individuals with Opioid Use Disorder	Substance Use and Misuse	[54]
29	Cashwell S.T., Campbell M., Cowser J.	Stone soup: social work community engagement in rural America’s opioid crisis	Social Work in Mental Health	[55]
30	Cotti C.D., Gordanier J.M., Ozturk O.D.	The relationship of opioid prescriptions and the educational performance of children	Social Science and Medicine	[56]
31	Barnes M.C., Kelly T.J., Piemonte C.M.	Demanding Better: A Case for Increased Funding and Involvement of State Medical Boards in Response to America’s Drug Abuse Crisis	Journal of Medical Regulation	[57]
32	Deshazer C., Dominic O., Deleo C., Johnson R.	Impact of a health system’s three-pronged strategy to address the opioid epidemic in de, pa, and wv, 2013–2017	Open Public Health Journal	[5]
33	Ho J.A., Rovzar A.O.	Preventing neonatal abstinence syndrome within the opioid epidemic: A uniform facilitative policy	Harvard Journal on Legislation	[58]

**Table 2:** Coding framework and results.

Node	Number of Coding	
01_Authors 01_Academics 02_Not academics/Practitioners 03_Collaboration between academics and practitioners	19 5 9	33
02_Geographic area 01_United States 02_Not localized 03_Canada 04_Multi-area	28 3 1 1	33
03_Type of pathology/department of reference 01_In global terms 02_Chronic pain 03_Emergency 04_Cardiology 05_Post operative pain 06_Neonatology/Pediatrics 07_Twin epidemic	19 1 3 1 1 5 8	33
04_Research method 01_Qualitative 02_Quantitative 01_Interview 01_Other 03_Literature Review	6 5 20 2	33
05_Type of impact 01_Social 01_Increase in deaths 02_Abuse of minors 03_Illegal use of methamphetamine, fentanyl, ecc. 04_Domestic violence 05_Homicide 06_Negative repercussions on academic performance 02_Economic 01_Increase in Medicaid enrollments 02_Increase in healthcare costs 03_Public health 01_Increase in ER accesses 02_Infectious Complications 03_Endiocarditis 04_Increase in the use of naloxone 05_Nonatal abstinence syndrome 06_Preterm birth 07_Sleep issues 04_Politics and justice 05_Professional training	24 1 9 2 3 1 1 8 2 2 1 1 2 1 1 13 1	33
06_Stakeholders 01_Patients 02_Health system/Pharmacists 03_Contributions 04_Minors 05_Childcare system 06_Justice 07_Families 08_Administrative employees 09_Communities 10_Schools 11_Financers 12_Social assistants 13_State Medical Commission	16 14 2 5 3 15 6 1 3 1 1 1 1	
07_Recommended strategies 01_Yes 01_Prevention enhancement 02_Medical assistance 03_Real-time data to deal with overdoses 04_Real-time data to manage outbreaks 05_Increase/improve naloxone prescriptions 06_Increase training 07_Syringe Service Programs 08_Opioid treatment programs 09_Involvement of political decision makers 10_More personalized care 11_Training of patients and families 12_Strengthening the collaboration between the health and safety sector 13_Improvement of childcare services 14_New best practices 15_Research/Financing 16_Multi-sector approaches 17_Wage increases 18_Increase in employment 19_Reduction in stereotypes 20_Increase access to health care 21_Reduction in over-policing 22_More investments in public health 23_Reduction in barriers 24_ Legalization of marijuana 25_Improve the relationship between pharmacist and general practitioner 26_Involvement of social workers 27_Better health check on possible opioid problems 28_Increase in the fight against drug dealing 02_No	8 5 3 5 5 11 2 6 3 3 4 1 1 3 6 5 1 1 1 1 2 3 1 1 1 1 2 3 0	

Source: Authors’ elaboration.

**Table 3:** Classification of the most relevant impacts.

Type of Impact	Most Relevant Subgroups	References	Extracted Contents
Social, Health and Public Health	1. Illegal use of methamphetamine-fentanyl-etc.	Eeckhaut, M.C.W., et al. 2020 [6]	“Synthetic opioids are now driving the US epidemic11 as prescription opioid is being replaced by heroin adulterated with fentanyl and its analogues.” “…prescription opioids are a “gateway” to other more dangerous or illegal opioids (e.g., heroin, illicitly manufactured fentanyl)…”
	2. Homicides	Rosenfeld, R., et al., 2020 [50] Testa, A., et al., 2021 [51]	“The results reveal a positive association between change over time in homicide and opioid-related deaths, net of multiple socioeconomic and demographic controls, in both the Non-Hispanic White and Black population.” “Study findings revealed a positive bivariate association between opioid mortality and homicide rates.”
	3. Increase in the number of deaths	Chiu, A.S., et al., 2018 [14] U.S. Department of Health and Human Services, Centers for Disease Control and Prevention, 2020 Seltzer, N., 2020 [39]	“As of 2014, the number of opioid-related deaths in the United States had risen to nearly 50,000 a year, a number now greater than the annual deaths from motor vehicle accidents.” “In 2017, a total of 70,237 persons in the United States died from a drug overdose, and 67.8% of these deaths involved an opioid recognized as a controlled/scheduled substance by the federal government.” Drug overdose deaths in the United States continued to rise (…) reducing overall life expectancy (…) trend in life expectancy that has not occurred in over a century.”
Medical	1. Infectious complications	U.S. Department of Health and Human Services, Centers for Disease Control and Prevention, 2017	“(…) infectious complications of intravenous drug use constitute a major cause of morbidity leading to hospitalization”
	2. Neonatal abstinence syndrome	Saunders, J.B., et al., 2018 [36]	“Opioid misuse during pregnancy is increasingly common and is associated with preterm birth and neonatal abstinence syndrome.”
Economic, politics and justice	1. Increase in health care costs	DeShazer, C., et al., 2020 [5] Ho, J.A., et al., 2017 [58]	“Employees with substance abuse disorder have three times more in health care costs of the average worker.” “The expenses of treating, monitoring, and rehabilitating these chemically dependent newborns are predominantly shouldered by state taxpayers and are extremely costly, with a mean cost per stay exceeding $90,000 for pharmacologically treated cases.”
	2. Violence and safety	Testa, A., et al., 2021 [51] Gollust, S.E., et al. 2021 [48] Saunders, J.B., et al., 2018 [36]	“The pharmacological effects of opioids may prompt users to become involved in illicit markets and the violence associated with such markets (…) Individuals who are physically dependent on opioids may also resort to economic-compulsive offending, which may include violence in the instance of robbery or drug dealing, to support their use.” “The opioid epidemic has had a profound effect on American public health, and studies suggest it has had a profound effect on American politics as well. Research suggests a relationship between the severity of the opioid crisis in a community and aggregate-level political behavior, including voting for Donald Trump in 2016 (…)” “State policymakers have made broad efforts to address the opioid epidemic at the public health and safety level (e.g., prescription drug monitoring programs) and the health care system level (…). Legislatures, governors, and state agencies have also taken specific steps to combat increasing trends in opioid misuse among pregnant women.”

**Table 4:** Main stakeholders.

Stakeholders	References	Extracted Contents
Patients	Chiu, A.S., et al., 2018 [14] Saunders, J.B., et al., 2018 [36] Seltzer, N., 2020 [39] Scorsone, K.L., et al., 2021 [54]	“(…) excess prescription of opioids present a hazard to both the patient and those around them.” “Strategies to address opioid misuse before pregnancy are efforts for the general population that also happen to include reproductive-age women.” “The drug epidemic continues to disrupt the lives of individuals, families, and communities throughout the country.” “That’s not how it works. You’re doing it because you have to because your body is telling you that you need it. Once you get that chemical imbalance in your brain, that’s it. You will always be an addict.”
Justice	Scorsone, K.L., et al., 2021 [54] Wagner, J., et al., 2021 [46]	“All of the participants described having lost employment income, leading to reliance on stealing from family and burglary.” “I broke into one house, and I got caught for it, because I was sloppy about it, but I admitted to it when the cops showed up, and I knew the people. I told them, “Yeah, I’m an addict. I needed to get my fix.” “(economic) disadvantage is a highly significant predictor of increased drug selling and drug possession arrests in a neighborhood (…). Drug possession arrests, in turn, are associated with higher neighborhood opioid overdose death rates (…).”
Healthcare System/Pharmacists	Rao, D., et al., 2021 [53]	“Oftentimes, pharmacists call prescribers and question long term use of medications and are met with trouble.” “Declining to fill a prescription based on clinical judgment (in absence of obvious red flags like early fills) is an uncomfortable concept for many pharmacists, I feel, because there is concern that it will damage patient and physician relationships by making you the “difficult” or “intrusive” pharmacist.
Families	Wentzlof, C.A., et al., 2021 [47]	“Research has established a strong, positive correlation between homicides and substance use and also between homicides and intimate partner violence.”
Minors	Queeneth, U., et al., 2019 [42] Lynch, S., et al., 2018 [34]	“Younger children are more vulnerable to the accidental ingestion of opioids. Certain strategies should be developed and also implemented to curb heroin overdoses in the pediatric population.” “In the past 18 years, nearly 8986 children and adolescents have died from illicit opioid use (…)” “The recent opioid epidemic has led to rising numbers of women who use opioids during pregnancy and infants born with neonatal abstinence syndrome (NAS), raising the question of whether there has been a consequent rise in the numbers of these infants reported to the child welfare system.”

## Data Availability

The datasets used and analyzed during the current study are available from the corresponding author upon reasonable request.
